# Trajectories of Health-related quality of life in patients with Advanced Cancer during the Last Year of Life: findings from the COMPASS study

**DOI:** 10.1186/s12904-022-01075-3

**Published:** 2022-10-14

**Authors:** Jonathan Lee, Mahham Shafiq, Rahul Malhotra, Semra Ozdemir, Irene Teo, Chetna Malhotra

**Affiliations:** 1 Lien Centre for Palliative Care, Duke-NUS Medical School, Singapore, Singapore; 2 Program in Health Services and Systems Research, Duke-NUS Medical School, Singapore, Singapore; 3 Centre for Ageing Research and Education, Duke-NUS Medical School, Singapore, Singapore; 4 Division of Supportive and Palliative Care, National Cancer Centre Singapore, Singapore, Singapore

**Keywords:** Cancer, Health-related quality of life, Neoplasms, Oncology, Palliative Care, Prospective study, Singapore, Surveys and questionnaires

## Abstract

**Background:**

Patients with advanced cancer prioritise health-related quality of life (HrQoL) in end-of-life care, however an understanding of pre-death HrQoL trajectories is lacking. We aimed to delineate and describe the trajectories of physical, social, emotional and functional HrQoL during last year of life among advanced cancer patients. We assessed associations between these trajectories and patient socio-demographic characteristics, healthcare use and place of death.

**Methods:**

We used data from 345 decedents from a prospective cohort study of 600 patients with a solid advanced cancer receiving secondary care at public hospitals in Singapore. Patients were surveyed every three months until death and HrQoL was assessed using the Functional Assessment of Cancer Therapy – General (FACT-G) questionnaire. Interviews were conducted between July 2016 and December 2019. Group-based multi-trajectory modelling was used to assess potential heterogeneity in the four HrQoL dimensions during patients’ last year of life.

**Results:**

We identified four distinct trajectories of HrQoL − (1) overall high HrQoL (47% of sample), (2) progressively decreasing HrQoL (32%), (3) asymmetric decline in HrQoL (13%), (4) overall low HrQoL (8%). Compared to patients with secondary or above education, those with primary education or less (β = 1.39, SE = 0.55, p-value = 0.012) were more likely to have “progressively decreasing HrQoL” or “overall low HrQoL” in contrast to “overall high HrQoL”. Compared to patients with ‘overall high HrQoL’, those with ‘overall low HrQoL’ had longer length of hospital stay during the last year of life (β = 0.47, SE = 0.21, p-value = 0.026) and were more likely to die in a hospice/care home (β = 1.86, SE = 0.66, p-value = 0.005).

**Conclusion:**

Our results showed heterogeneity in deterioration of HrQoL among patients with advanced cancer in the last year of life. Systematic monitoring of HrQoL, early identification and referral of high-risk patients to palliative care may provide timely relief and mitigate the steep decline in their HrQoL.

**Trial Registration:**

: NCT02850640.

**Supplementary Information:**

The online version contains supplementary material available at 10.1186/s12904-022-01075-3.

## Introductionall reference citations should be inside a bracket.

Cancer is a global health concern. There were about 19.3 million new cancer cases and 10 million deaths due to cancer in 2020. These figures are expected to almost double by 2040 [1], illustrating a high mortality rate despite better treatment, and emphasizing the importance of palliative care for patients with an advanced cancer. The World Health Organization recognizes palliative care as a human right to health [2], which seeks to maximize health-related quality of life (HrQoL) in multiple dimensions like physical, functional, social and psychological at the end of life [3]. Studies have found that at the end of life, patients with advanced cancer prioritize HrQoL above life extension [4,5] and that palliative care can improve their HrQoL [6–9]. A clear understanding of HrQoL trajectories at the end of life among patients with advanced cancer can ensure prompt and targeted palliative care interventions.

Previous studies have described that HrQoL of patients with advanced cancer declines steeply during the last six months of their life [10–12]. Yet, several gaps remain. First, many studies were limited to the last six months of patients’ life or less, potentially missing critical deteriorations prior to that period. Second, studies were limited to either one type of cancer or used only one data point for each patient [13,14]. Third, a few studies only focused on patients already receiving palliative care, resulting in a selection bias [10–12, 15]. Fourth, the aforementioned studies only focused on describing an average trajectory of HrQoL among patients. Given differences in patient demographics, access to health care and ability to cope, there is likely heterogeneity in patients’ HrQoL trajectories, which has not yet been elucidated.

To address the above gaps in our understanding of HrQoL at the end of life, we conducted a prospective longitudinal study among patients with solid metastatic cancers. Our primary aims were to describe the heterogeneity in joint trajectories of physical, social, emotional and functional HrQoL during last year of life, and to identify patient socio-demographic characteristics that predict membership of the delineated trajectories. Studies have shown that females [16] and patients with low socioeconomic status (SES) [17] report greater impairments in their HrQoL [18]. Older patients report better overall HrQoL compared to younger patients despite suffering from comorbidities [19]. Hence, we hypothesize that females and patients with low educational attainment (indicative of low SES [20–22]) will be more likely to belong to worse HrQoL trajectories and older patients will be more likely to belong to trajectories representing better HrQoL.

To further explore differences across the joint HrQoL trajectories, we also assessed if healthcare use during the last year of life and place of death varied across the delineated joint HrQoL trajectories. Studies have shown that patients who experience worse HrQoL have longer length of hospital stay [23] and are more likely to die in a hospital compared to home or hospice [24]. Hence, we hypothesize that patients with trajectories indicative of worse HrQoL will be associated with more hospital admissions, emergency department (ED) visits, longer length of hospital stay, and be more likely to die in the hospital.

## Methods

### Study design

Data used in this study is from the Cost of Medical Care of Patients with Advanced Serious Illness in Singapore (COMPASS) study. This is a prospective cohort study of 600 patients diagnosed with stage IV solid malignancy, surveyed every three months until death.

### Participants

Between July 2016 and March 2018, the COMPASS study recruited 600 patients from outpatient medical oncology clinics at two major public hospitals in Singapore. Patient inclusion criteria were diagnosis of stage IV solid cancer, age 21 years or above, Singapore citizen or permanent resident, cognitively able to answer the survey (determined through medical records or Abbreviated Mental Test [25] administered to participants ≥ 60 years), and Eastern Cooperative Oncology Group performance status ≤ 2 [26]. Eligible patients who were willing to participate provided written informed consent. The SingHealth Centralised Institutional Review Board (2015/2781) approved the study. Protocol details have been published elsewhere [27].

### Study variables

HrQoL: The Functional Assessment of Cancer Therapy – General (FACT-G V4) questionnaire was used to assess four dimensions of HrQoL: physical (7 items), functional (7 items), emotional (6 items) and social/family (7 items) well-being [28]. Responses to each question were rated on a five-point scale, as not at all (= 0), a little bit (= 1), somewhat (= 2), quite a bit (= 3) and very much (= 4). The score for each HrQoL dimension was calculate; higher score indicating better HrQoL in that dimension. Total score for each dimension range from physical 0–28; social 0–28, functional 0–28; and emotional 0–24. English and Chinese versions of FACT-G were obtained by the developer [29] while the Malay version was translated and back-translated according to developer guidelines to ensure comparability with the English version.

Patient socio-demographics: Age, gender, marital status and highest educational attainment (primary or lower, secondary, above secondary) were obtained from patients’ baseline survey.

Health care utilization: Number of hospital admissions, total length of hospital stay, and number of ED visits during patients’ last year of life were determined using billing records.

Place of death: Information on patients’ place of death was obtained from medical records and caregiver self-reports.

### Statistical analysis

Analysis was performed using data of patients who died between September 2016 and December 2019, and who had responded to at least one patient survey during their last year of life.

Group-based multi-trajectory modelling was used to assess potential heterogeneity in the four HrQoL dimensions during patients’ last year of life. Group-based multi-trajectory modelling is a form of group-based trajectory modelling that defines joint trajectories for multiple related outcomes thereby allowing us to jointly model the four dimensions. [30].

We modelled the physical, functional, emotional and social HrQoL dimensions assuming a censored normal distribution and identity link. For determining the optimal number of joint trajectories in the patient sample, we varied the number of joint trajectories and the polynomial function (i.e., quintic, quartic, cubic, quadratic, linear intercept) for each HrQoL dimension. For each specified number of joint trajectories, the best-fitting polynomial function for each HrQoL dimension was derived by first specifying a quintic (order of 5) polynomial function, and sequentially moving down by an order of one if the specified function was statistically not significant. We retained the highest order which was significant for the corresponding HrQoL dimension, otherwise the intercept (zero-order) function was retained regardless of significance. Patients were then assigned an exclusive joint trajectory based on their highest posterior probability of membership. Up to six joint trajectories were tested.

In selecting the optimal number of joint trajectories, we aimed to minimize the Bayesian Information Criterion (BIC), with minimum thresholds for the joint trajectory membership probability (at least 5%), value of average posterior probability (at least 0.7), and odds of correct classification (at least 5).

We then assessed if patient socio-demographic characteristics (e.g. age, gender, highest educational attainment) predicted membership of the selected delineated joint trajectories via multinomial logistic regression.

In addition, we assessed associations of the selected joint trajectories (as independent variable) with number of hospital admissions, total length of hospital stay, number of ED visits and place of death (as outcome variables). We used negative binomial regression for number of hospital admissions and total length of hospital stay during the last year of life, logistic regression for more than one ED visit during last year of life, and multinomial logistic regression for place of death (home/hospice or care home/hospital). All models were adjusted for patients’ age at death, gender and highest educational attainment.

We ran a sensitivity analysis using subsample of patients with no missing surveys since recruitment, and no missing surveys in 12 months prior to death. We conducted group-based multi-trajectory modelling using the same approach as above and compared the number and shape of selected joint trajectories, as well as the proportion of patients classified into similar trajectories between the main and sensitivity analysis.

All analyses were conducted using Stata version 16.

## Results

Of the 600 patients who consented and participated in the COMPASS study, 354 (59%) patients died during the study period. Among them, 9 (3%) patients did not complete at least one survey during the last year of life. Thus, the analysis cohort for this study consists of the remaining 345 (97%) patients. Patients in the analysis cohort, compared to the 246 patient who were still alive (41%), were more likely to be males and with gastrointestinal or genitourinary/ gynaecologic cancers. The two groups did not differ by age.

Table 1 describes patient socio-demographics, health care utilization and place of death. Average age of patients at the beginning of last year of life was 61 years (standard deviation (SD) = 10.6 years), with slightly more than half being males (51%). Most were married and possessed highest educational attainment of secondary school or higher. In the last year of life, patients had an average of 3.2 hospital admissions (SD = 2.3) and were hospitalized for an average of 23.3 days (SD = 21.7). Slightly more than half (51%) of the patients reported having more than 1 ED visit. Most patients (62%) died in the hospital, while the rest died at home (25%) or in a hospice/care home (13%).

**Table 1:** Patient characteristics

	N = 345
**Demographic characteristics**	
Age at beginning of last year of life, mean (SD) (Range: 25–88)	61 (10.6)
≤ 45 years of age, n (%)	30 (8.7)
46–65 years of age, n (%)	202 (58.5)
≥ 66 years of age, n (%)	113 (32.8)
Gender, n (%)	
Male	175 (50.7)
Female	170 (49.3)
Marital status, n (%)	
Married	248 (71.9)
Separated/Widowed/Divorced/Never married	97 (28.1)
Highest educational attainment, n (%)	
Primary or lower	147 (42.6)
Secondary	113 (32.8)
Above secondary	85 (24.6)
**Health related quality of life dimensions at the beginning of the last year of life**	
Social (Range: 2–28), mean (SD)	21.7 (5.1)
Physical (Range: 1–28), mean (SD)	22.3 (5.7)
Functional (Range: 2–28), mean (SD)	19.4 (6.1)
Emotional (Range: 0–24), mean (SD)	19.3 (4.5)
**Healthcare utilization during last year of life** ^**a**^	
Number of hospital admissions (Range: 0–14; Median: 3), mean (SD)	3.2 (2.3)
Total length of hospital stay (Range: 0–122; Median: 18), mean (SD)	23.3 (21.7)
Had more than 1 emergency department visit, n (%)	170 (51.0)
**Place of death** ^**b**^	
Hospital, n (%)	167 (62.3)
Home, n (%)	67 (25.0)
Hospice/ Care home, n (%)	34 (12.6)

^a^ using subsample of participants who died before October 2019 (N = 333)

^b^ using subsample of participants with available information on place of death (N = 268)

^†^ SD: Standard deviation

### Joint trajectories

**Fig. 1:**
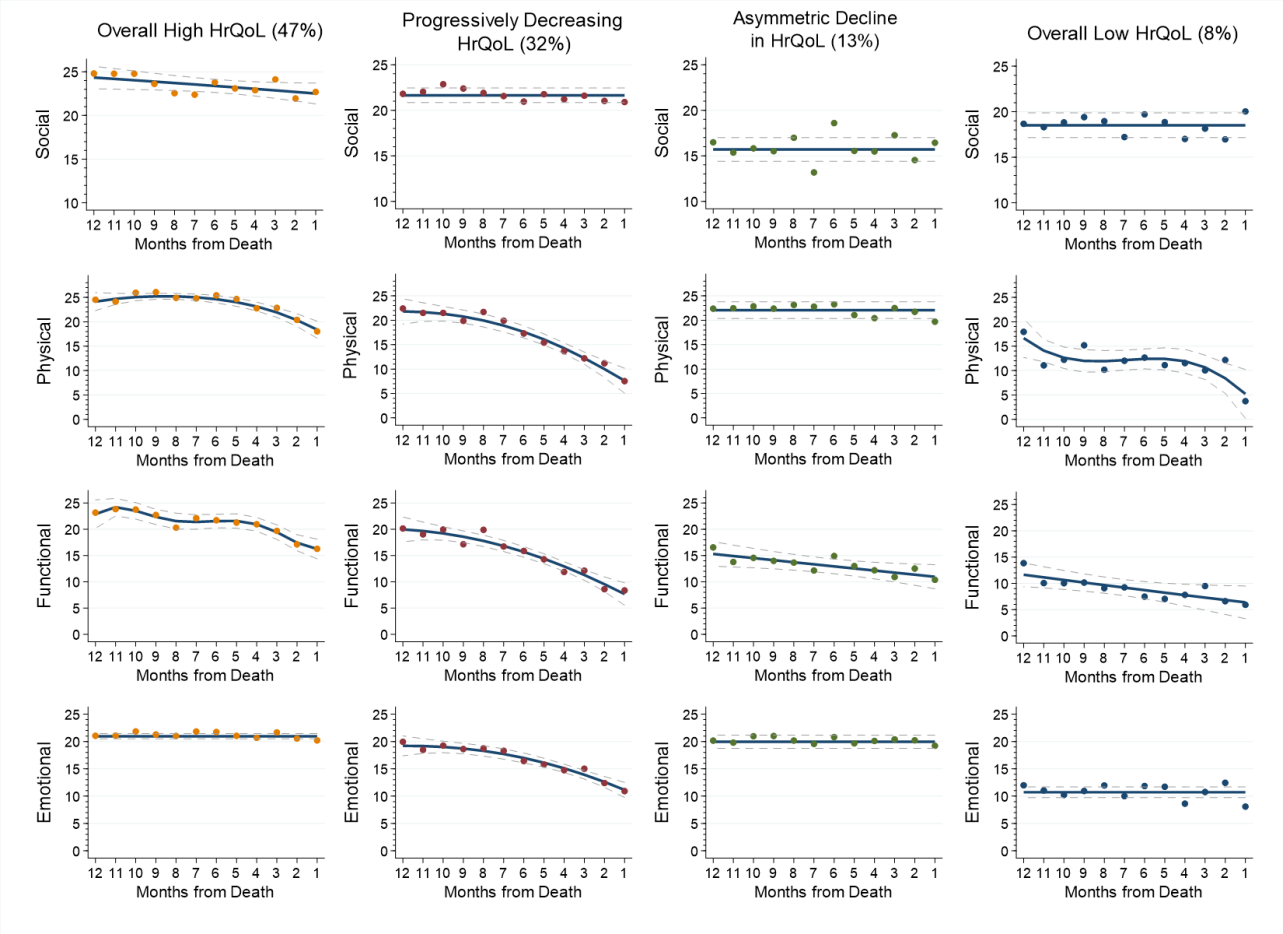
Joint trajectories of social, physical, functional and emotional health related quality of life (HrQoL) during last year of life (N = 345)

In determining the optimal number of joint HrQoL trajectories, we fitted 43 models (with no covariates) and selected the model with four joint trajectories. While its BIC was not the closest to zero (-11,680), it was marginally higher (0.07%) than the BIC for the five-group model. The trajectory membership probability ranged between 8 and 47%, and the average posterior probabilities ranged between 0.86 and 0.94. Based on the observed pattern over time, we named these distinct joint trajectories as (1) overall high HrQoL, (2) progressively decreasing HrQoL, (3) asymmetric decline in HrQoL, and (4) overall low HrQoL (Fig. 1).

Patients with “overall high HrQoL” trajectory (47%) reported highest levels for all dimensions at the onset of the last year of life, compared to other joint trajectories. While social, physical and functional HrQoL experienced slight declines, emotional HrQoL remained high throughout the last year of life.

Patients with “progressively decreasing HrQoL” (32%) reported high levels for all dimensions at the onset of the last year of life. Although physical, functional and emotional HrQoL declined considerably, social HrQoL remained high throughout the last year of life.

Patients with “asymmetric decline in HrQoL” (13%) reported consistently high levels of physical and emotional HrQoL throughout the last year of life. Functional HrQoL was low at onset and steadily declined throughout the last year of life, while social HrQoL was consistently at the lowest level across all joint trajectories.

Patients with “overall low HrQoL” (8%) reported moderate levels of social and physical HrQoL coupled with relatively low levels of functional and emotional HrQoL at the beginning of last year of life. Social and emotional HrQoL remained consistently low while physical and functional HrQoL declined to the lowest levels across all joint trajectories. Deterioration was constant for functional HrQoL but more marked in the first and last quarters of the year for physical HrQoL.

Predictors of membership.

Having identified the joint trajectories, we assessed age, gender, marital status and highest educational attainment at 5% level of significance (Table 2).

**Table 2:** Predictors of membership of the joint trajectories (N = 345)

	Joint Trajectories (Reference: Overall High HrQoL)			
	**Overall High HrQoL**	**Progressively Decreasing HrQoL**	**Asymmetric Decline in HrQoL**	**Overall Low HrQoL**
		**Coef. (Std. Err.)**	**Coef. (Std. Err.)**	**Coef. (Std. Err.)**
**Age at beginning of last year of life**				
≤ 45 years of age		1.14 (1.19)	3.69* (1.79)	2.06 (1.21)
46–65 years of age		-0.62 (0.51)	1.97 (1.32)	0.09 (0.55)
≥ 66 years of age *[Reference]*				
**Gender**				
Male *[Reference]*				
Female		-0.54 (0.49)	-1.36* (0.69)	-0.75 (0.52)
**Marital status**				
Married *[Reference]*				
Separated/Widowed/ Divorced/Never married		-0.19 (0.48)	0.47 (0.68)	-0.40 (0.51)
**Highest educational attainment**				
Primary or lower		1.39* (0.55)	-1.00 (1.22)	1.20* (0.58)
Secondary		0.82 (0.53)	0.89 (0.71)	0.72 (0.56)
Above secondary *[Reference]*				

* Statistically significant at the 5% level

^†^ HrQoL: Health-related Quality of Life; Coef.: Coefficient; Std. Err: Standard Error

Patients with primary or lower educational attainment, versus higher education, had a higher likelihood of belonging to the “progressively decreasing HrQoL” trajectory (β = 1.39, SE = 0.55, p-value = 0.012) and the “overall low HrQoL” (β = 1.20, SE = 0.58, p-value = 0.039) than the “overall high HrQoL” trajectory.

Patients aged ≤ 45 years, versus > 66 years, had a higher likelihood (β = 3.69, SE = 1.79, p-value = 0.039), while females had a lower likelihood (β=-1.36, SE = 0.69, p-value = 0.049) of belonging to the “asymmetric decline in HrQoL” trajectory than the “overall high HrQoL” trajectory.

Patient aged 46–65 years (versus > 66 years), those with secondary education (versus higher education), and their marital status were not statistically significant in predicting membership of the joint trajectories.

### Associations with distal outcomes

Using appropriate distribution models, we evaluated the joint trajectories for associations with healthcare use and place of death at 5% level of significance (Table 3).

**Table 3:** Association^a^ of the delineated trajectories with healthcare utilization during the last year of life and place of death ^b^

	Number of hospital admissions 1 (Range: 0–14; Median: 3) (N = 333)		Total length of hospital stay 1 (Range: 0–122; Median: 18) (N = 333)		More than 1 emergency department visit 2 (N = 333)		Place of death [Ref: Hospital] 3 (N = 268)			
							**Home**		**Hospice/ Care home**	
	**Coef. (Std. Err.)**	**[95% CI]**	**Coef. (Std. Err.)**	**[95% CI]**	**Coef. (Std. Err.)**	**[95% CI]**	**Coef. (Std. Err.)**	**[95% CI]**	**Coef. (Std. Err.)**	**[95% CI]**
Overall High HrQoL *[Reference]*										
Progressively Decreasing HrQoL	0.01 (0.09)	[-0.16, 0.17]	0.01 (0.12)	[-0.23, 0.25]	-0.22 (0.26)	[-0.72, 0.29]	0.48 (0.34)	[-0.19, 1.16]	0.77 (0.54)	[-0.28, 1.82]
Asymmetric Decline in HrQoL	-0.29* (0.13)	[-0.55, -0.04]	-0.22 (0.17)	[-0.56, 0.12]	-0.60 (0.36)	[-1.31, 0.11]	0.83 (0.46)	[-0.07, 1.74]	1.68* (0.55)	[0.61, 2.76]
Overall Low HrQoL	0.09 (0.14)	[-0.19, 0.37]	0.47* (0.21)	[0.06, 0.89]	0.02 (0.43)	[-0.83, 0.87]	-0.64 (0.81)	[-2.23, 0.95]	1.86* (0.66)	[0.57, 3.16]

* Statistically significant at the 5% level

^1^ Negative binomial regression, 2 Logistic regression, 3 Multinomial logistic regression

^a^ All estimates were adjusted for age at beginning of last year of life, gender, marital status and highest educational attainment

^b^ Subsample of participants who died before October 2019

^†^ Coef.: Coefficient; Std. Err: Standard Error, CI: Confidence Interval; HrQoL: Health-related Quality of Life

Patients with “asymmetric decline in HrQoL” trajectory were associated with fewer hospital admissions (β=-0.29, SE = 0.13, p-value = 0.025) and were more likely to die in a hospice/care home (β = 1.68, SE = 0.55, p-value = 0.002) relative to those with “overall high HrQoL”.

Patients with “overall low HrQoL” trajectory were associated with longer hospital stay (β = 0.47, SE = 0.21, p-value = 0.026) and were more likely to die in a hospice/care home (β = 1.86, SE = 0.66, p-value = 0.005) relative to those with “overall high HrQoL”.

### Complete case sensitivity analysis (n = 207)

The 5-group model was selected despite not yielding BIC closest to zero (-7594) as the difference in BIC from a 6-group model was marginal (0.16%). Average posterior probability of joint trajectory membership ranged between 0.87 and 0.99. Eighty-five percent of patients in this analysis belonged to similar trajectory groups as the main analysis.

## Discussion

### Main findings and results

This prospective study identified joint trajectories of various dimensions of HrQoL during the last year of life among patients with advanced cancer, and their relationship with patient demographics and healthcare use. Four distinct joint trajectories were identified – overall high HrQoL; progressively decreasing HrQoL; asymmetric decline in HrQoL; and overall low HrQoL.

Results revealed that close to half of our patient sample (47%) had a ‘overall high HrQoL’ trajectory, with slight declines in social, physical and functional HrQoL, which were high to start off with, and constantly high emotional HrQoL during the last year of life. This is inconsistent with previous studies that demonstrated rapid decline in HrQoL during the final months of life [10,11,13,14]. Given that Singapore ranks 12th (amongst 80 countries) on the 2015 Quality of Death Index [31], coupled with efforts by the government to raise palliative care standards [32], it is possible that a steep decline in HrQoL during final months of life was mitigated for many patients.

The remaining patients were likely to report a lower HrQoL at the beginning of the last year of life or experience greater declines in one or more subscales. In particular, patients with ‘overall low HrQoL” experienced marked declines in physical HrQoL during the first and last quarters of the year as well as constant decline in functional HrQoL over the year, but had consistently moderate social and emotional HrQoL. In line with our hypothesis, patients having this joint trajectory were more likely to have low educational attainment (indicative of low SES [20–22]), which has been shown to be associated with comparatively poorer physical and mental health [33–35]. Previous studies have shown that patients from lower SES have more difficulties coping with their illness, lack awareness regarding palliative services, and are referred late to the services. Caregivers of these patients are also less prepared for end-of-life care, resulting in many patients dying in the hospital despite wanting a home death. In Singapore, one in four Singaporeans die at home despite 77% wanting to do so [36]. With the government committed to reducing the proportion of people dying in hospitals from 61–51% by 2027 [37,38], timely referral to palliative care services may be beneficial in maintaining these patients’ HrQoL [6–9].

Our results showed that younger patients (aged ≤ 45 years) were more likely have the trajectory representing ‘asymmetric decline in HrQoL’. These results are largely consistent with existing literature showing that during the last year of life, younger patients experience higher physical HrQoL but lower social and functional HrQoL compared to older adults [12,39]. However, our finding is contradictory to reports showing association of older age with better emotional HrQoL amongst advanced cancer patients [40–42]. We also found that female patients were less likely to belong to this group, which could be a result of women possessing a larger social network than men, which has been shown to be associated with better social HrQoL [43,44].

Consistent with our hypothesis, patients with decline in HrQoL across all dimensions (‘overall low HrQoL’) had the longest hospital stay in the last year of life. Previous studies have shown that decline in HrQoL is related to high symptom burden among patients [10,45], which in turn results in higher ED visits, hospital admissions, and prolonged hospitalizations [46]. At the same time, longer hospitalizations often lead to increased functional disability, decreased emotional HrQoL and cognitive decline [47–50]. Hence, the relationship between healthcare use and HrQoL can be bidirectional. Regardless, interventions to screen and treat patients at risk of being in this trajectory can potentially reduce the decline in patients’ HrQoL, and also reduce their health care utilization.

Patients having the worst social HrQoL (‘asymmetric decline in HrQoL’ group, constituting 13% of the sample) had fewer hospital admissions – a reflection of their high physical HrQoL. It is also possible that this group of patients may not have enough social support to be cared for at home, which could be related to their lower SES [51], thereby increasing their chances of being referred to a hospice and dying there.

Our results suggest that among patients at risk of dying within one year, those aged ≤ 45years and belonging to lower SES, with longer hospital stays and multiple hospital admissions in the last year of life are vulnerable to poorer HrQoL. We recommend that these patients should be routinely and comprehensively assessed for various dimensions of HrQoL. Early and systematic referral to palliative care for these patients can prevent steep declines in their HrQoL by relieving symptom burden, improving psychological and social distress, as well as reducing hospitalization frequency and length of hospital stay [8,9,52].

### Strengths and weaknesses

There are several limitations to our study. First, HrQoL was only assessed every three months. Greater precision on the trajectories could be achieved with more frequent assessments. Second, the sample size used to assess association of healthcare use and place of death was reduced due to lack of data for participants who died between October and December 2019. Third, 14% of patient data was missing during the last year of life. However, we used the full-information maximum likelihood (FIML) method for managing the missing data [53–55]. Fourth, refusal of patients to participate in the study due to poor health could be a potential source of selection bias. However, we expect this bias to be low given that the non-response rate in the study was low (38%) and that the main reason for patients to refuse participation was lack of interest (79%) rather than ill-health (9%).

A key strength of this study is the use of prospective longitudinal data with a large sample of patients with advanced solid cancer. First, it addresses gaps in previous studies, which used shorter study periods that risked missing critical deteriorations [10–12] or relied on caregiver reports that compromised accuracy [56,57]. Third, we recruited patients from two large public hospitals that together see more than 70% of all cancer cases in Singapore [58]. Lastly, unlike previous studies, our study examined the heterogeneity in the various dimensions of HrQoL instead of only looking at the overall average HrQoL trajectory or the trajectories for each dimension separately.

## Conclusion

In conclusion, our study described four joint trajectories of HrQoL dimensions among patients with advanced cancer in the last year of life. Results showed that patients aged ≤ 45years, those from lower SES, with longer hospital stays and multiple hospital admissions experienced worse or rapid deterioration in HrQoL during their last year of life. Systematic monitoring of HrQoL, early identification and referral of high-risk patients to palliative care may provide timely relief and mitigate the steep decline in their HrQoL.

## Electronic supplementary material

Below is the link to the electronic supplementary material.

Supplementary Material 1

Supplementary Material 2

Supplementary Material 3

Supplementary Material 4

Supplementary Material 5

## Data Availability

The datasets generated during and/or analysed during the current study are not publicly available as their access requires approval from institutional review board. The datasets are available from the corresponding author on reasonable request. Every request will be reviewed by the approving institutional review boards and the researcher will need to sign a data access agreement with National University of Singapore after approval.

## List of abbreviations

BIC: Bayesian Information Criterion
COMPASS: Cost of Medical Care of Patients with Advanced Serious Illness in Singapore
ED: Emergency Department
FACT-G: Functional Assessment of Cancer Therapy – General
FIML: Full-Information Maximum Likelihood
HrQoL: Health-related Quality of Life
SD: Standard Deviation
SES: Socioeconomic Status
