# Supplementary material for: Trajectories of Health-related quality of life in patients with Advanced Cancer during the Last Year of Life: findings from the COMPASS study
Source: BMC Palliat Care. 2022 Oct 14;21:183. doi: 10.1186/s12904-022-01075-3 (PMC9569120; doi:10.1186/s12904-022-01075-3)
Supplement: Supplementary file 1 — Supplementary Material 1 [file 12904_2022_1075_MOESM1_ESM.docx]

**Supplementary Figure 1. COMPASS participant recruitment flow diagram**

Patients approached (n=1,137)

Ineligible patients (n=95)

- Did not meet inclusion criteria

Eligible patients (n=1,042)

Declined to participate (n=393)

- Not interested/ Indecisive (n=306)
- No time to participate (n=4)
- Research fatigue (n=20)
- Too ill or has hearing, vision, or speech impairment (n=34)

Patients consent and enrolled (n=649)

Ineligible patients (n=95)

- Did not meet inclusion criteria

Baseline data collected (n=647)

- Records review only (n=47)
- Survey and records review (n=600)

Analysed (n=345)

- Excluded from analysis (n=302)
- Did not answer survey on the last year of life (n=9)
- Records review only (n=47)
- Still alive (n=246)
