# Supplementary material for: Trajectories of Health-related quality of life in patients with Advanced Cancer during the Last Year of Life: findings from the COMPASS study
Source: BMC Palliat Care. 2022 Oct 14;21:183. doi: 10.1186/s12904-022-01075-3 (PMC9569120; doi:10.1186/s12904-022-01075-3)
Supplement: Supplementary file 2 — Supplementary Material 2 [file 12904_2022_1075_MOESM2_ESM.docx]

**Supplementary Table 1. Functional form analyses for joint trajectory model selection**

|  | **Number of joint trajectories** | | | | |
| --- | --- | --- | --- | --- | --- |
|  | **2-Group** | **3-Group** | **4-Group** | **5-Group** | **6-Group** |
| **Main analysis (n=345)** |  |  |  |  |  |
| Bayesian Information Criterion (BIC) | -11860 | -11770 | -11680 | -11672 | -11645 |
| % Change in BIC (Group-on-Group) | - | 0.76% | 0.76% | 0.07% | 0.24% |
| Average posterior probabilities^a^ | 0.95 to 0.97 | 0.92 to 0.95 | 0.86 to 0.94 | 0.79 to 0.92 | 0.76 to 0.94 |
| Odds of correct classifications^b^ | 23.7 to 26.8 | 16.3 to 139.4 | 17.1 to 186.1 | 12.1 to 129.6 | 9.3 to 204.8 |
| **Complete case sensitivity analysis (n=207)** | |  |  |  |  |
| Bayesian Information Criterion (BIC) | -7758 | -7680 | -7650 | -7594 | -7582 |
| % Change in BIC (Group-on-Group) | - | 1.01% | 0.40% | 0.72% | 0.16% |
| Average posterior probabilities^a^ | 0.96 to 0.97 | 0.95 to 1.00 | 0.87 to 1.00 | 0.93 to 0.99 | 0.81 to 0.96 |
| Odds of correct classifications^b^ | 22.2 to 38.6 | 19.0 to 6389.2 | 22.3 to 3667 | 16.0 to 2594.3 | 10.3 to 6401.2 |

* Chosen models for the respective analyses are highlighted in grey
