# Supplementary material for: Trajectories of Health-related quality of life in patients with Advanced Cancer during the Last Year of Life: findings from the COMPASS study
Source: BMC Palliat Care. 2022 Oct 14;21:183. doi: 10.1186/s12904-022-01075-3 (PMC9569120; doi:10.1186/s12904-022-01075-3)
Supplement: Supplementary file 3 — Supplementary Material 3 [file 12904_2022_1075_MOESM3_ESM.docx]

**Supplementary Table 2. Cross tabulation of main analysis with complete case sensitivity analysis**

|  | | **Complete Case Sensitivity Analysis (n=207)** | | | | | |  |
| --- | --- | --- | --- | --- | --- | --- | --- | --- |
|  |  | **Group 1** | **Group 2** | **Group 3** | **Group 4** | **Group 5** | **Incomplete responses** | **No. in the same group** |
|  |  | **n (% of group)** | **n (% of group)** | **n (% of group)** | **n (% of group)** | **n (% of group)** |  |  |
| **Main Analysis**  **(n=345)** | Overall High HrQoL | 13 (100%) | 92 (87%) | 0 | 0 | 0 | 57 | 92 |
|  | Progressively Decreasing HrQoL | 0 | 6 (6%) | 57 (93%) | 0 | 0 | 46 | 57 |
|  | Asymmetric decline in HrQoL | 0 | 8 (7%) | 3 (5%) | 15 (100%) | 0 | 19 | 15 |
|  | Overall Low HrQoL | 0 | 0 | 1 (2%) | 0 | 12 (100%) | 16 | 12 |
|  | **Total** | 13 | 106 | 61 | 15 | 12 | - | **85%**  **(176/207)** |
