# Supplementary material for: Trajectories of Health-related quality of life in patients with Advanced Cancer during the Last Year of Life: findings from the COMPASS study
Source: BMC Palliat Care. 2022 Oct 14;21:183. doi: 10.1186/s12904-022-01075-3 (PMC9569120; doi:10.1186/s12904-022-01075-3)
Supplement: Supplementary file 4 — Supplementary Material 4 [file 12904_2022_1075_MOESM4_ESM.docx]

**Supplementary Table 3. Patient characteristics comparison of analysis cohort (N=345) with patients who were still alive (N=246)**

|  | **Analysis Cohort**  **(N=345)** | **Excluded Participants**  **(N=246)** | **p-value** |
| --- | --- | --- | --- |
| **Age, mean(SD)^** | 60.8 (10.6) | 60.1 (10.8) | 0.425 |
| **Gender, n(%)~** | | | |
| Male | 175 (50.7) | 100 (40.6) | 0.016 |
| Female | 170 (49.3) | 146 (59.3) |  |
| **Type of cancer, n(%)~** | | | |
| Breast | 55 (15.9) | 38 (15.4) | 0.043 |
| Gastrointestinal | 103 (29.9) | 52 (21.1) |  |
| Genitourinary/ Gynaecologic | 62 (18.0) | 67 (27.2) |  |
| Respiratory | 98 (28.4) | 69 (28.0) |  |
| Others* | 27 (7.8) | 20 (8.1) |  |

^t-test was used; ~chi-square test was used to test for statistical difference between the groups

*Includes head and neck, musculoskeletal, neurologic, skin and unknown cancer types
