# Supplementary material for: Trajectories of Health-related quality of life in patients with Advanced Cancer during the Last Year of Life: findings from the COMPASS study
Source: BMC Palliat Care. 2022 Oct 14;21:183. doi: 10.1186/s12904-022-01075-3 (PMC9569120; doi:10.1186/s12904-022-01075-3)
Supplement: Supplementary file 5 — Supplementary Material 5 [file 12904_2022_1075_MOESM5_ESM.docx]

**COMPASS Study Group**

Eric A Finkelstein^1,2^, Alethea Chung Pheng Yee^1,3^, Rebecca A. Dent^3^, Ravindran Kanesvaran^3^, Grace Meijuan Yang^3^, Nesaretnam Barr Kumarakulasinghe^4^, Noreen Chan^4^, Patricia Soek Hui Neo^4^, Yin Bun Cheung^5^, Huei Yaw Wu^6^, Hum Yin Mei Allyn^6^, Soh Mun Chin^7^, Richard Harding^8^, Lee Lai Heng^9^

^1^Lien Centre for Palliative Care, Duke-NUS Medical School, Singapore, Singapore

^2^Program in Health Services and Systems Research, Duke-NUS Medical School, Singapore, Singapore

^3^National Cancer Centre, Singapore, Singapore

^4^National University Cancer Institute, National University Hospital, Singapore, Singapore

^5^Centre for Quantitative Medicine, Duke-NUS Medical School, Singapore, Singapore,

^6^Tan Tock Seng Hospital, Singapore, Singapore

^7^Dover Park Hospice, Singapore, Singapore

^8^King’s College London, Cicely Saunders Institute, London, UK

^9^Singapore General Hospital, Singapore, Singapore
